# Restrictions on Hospital Referrals from Long-Term Care Homes in Madrid and COVID-19 Mortality from March to June 2020: A Systematic Review of Studies Conducted in Spain

**DOI:** 10.3390/epidemiologia4020019

**Published:** 2023-06-06

**Authors:** Maria Victoria Zunzunegui, François Béland, Fernando J. García López

**Affiliations:** 1 École de Santé Publique, Université de Montréal, Montréal, QC H3N 1X9, Canada; 2 National Epidemiology Centre, Instituto de Salud Carlos III, 28929 Madrid, Spain; 3 Networked Biomedical Research Center for Neurodegenerative Diseases (CIBERNED), 28029 Madrid, Spain

**Keywords:** triage, older adults, mortality, long-term care, hospitalization, COVID-19, Spain

## Abstract

In March 2020, a ministerial directive issued by the Government of the Community of Madrid (CoM) in Spain included disability-based exclusion criteria and recommendations against hospital referral of patients with respiratory conditions living in long-term care homes (LTCHs). Our objective was to assess whether the hospitalization mortality ratio (HMR) is greater than unity, as would be expected had the more severe COVID-19 cases been hospitalized. Thirteen research publications were identified in this systematic review of mortality by place of death of COVID-19-diagnosed LTCH residents in Spain. In the two CoM studies, the HMRs were 0.9 (95%CI 0.8;1.1) and 0.7 (95%CI 0.5;0.9), respectively. Outside of the CoM, in 9 out of 11 studies, the reported HMRs were between 1.7 and 5, with lower 95% CI limits over one. Evaluation of the disability-based triage of LTCH residents during March–April 2020 in public hospitals in the CoM should be conducted.

## 1. Introduction

### 1.1. The First Two Months of the COVID-19 Pandemic in Long-Term Care Homes in the Community of Madrid (Spain)

In the first two months (March and April 2020) of the COVID-19 pandemic, a disproportionately large number of COVID-19 deaths occurred in long-term care homes (LTCHs) in the Community of Madrid (CoM) (Spain). More specifically, in the region’s 470 LTCHs, with a reported 51,938 places, 9468 deaths occurred among LTCH residents; 7290 of those deaths occurred at the LTCH and 2178 at the corresponding referral hospital, totaling a mortality of 18.3% in these two months, if full occupancy is assumed. Cause of death was not available for those who died after transfer to the referral hospital. Of the 7290 deaths that occurred in LTCHs, 1118 (15.3%) were confirmed as COVID-19 deaths, 4676 (64.1%) as deaths with COVID-19 symptoms, and 1496 (20.5%) as deaths due to other causes [1].

### 1.2. The Context of Healthcare for the LTCH Population in Spain in 2020

LTCHs in Spain are not healthcare institutions. Residents, as any citizen of Spain, receive healthcare from the Spanish National Health System, including primary care at the local health center and hospital care at their LTCH’s referral hospital. Policies, funding, and standards concerning the healthcare system, public health services, and LTCHs are decided by regional governments. According to CoM legislation in 2020, LTCHs with more than 49 places were required to have an infirmary unit. For homes with more than 99 places, infirmary beds had to equal at least 5% of the total number of places, and a mortuary and an area to provide special services to residents, such as social work and rehabilitation, were required. Medical doctors were not required on the LTCH staff.

### 1.3. CoM Health Policy on LTCHs during the First Two Months of the COVID-19 Pandemic

In March and April of 2020, public hospitals in the CoM were near collapse [2]. During the third week of March, COVID-19 patients occupied almost 50% of acute hospital beds, and by the end of March occupancy was over 100%.

On 12 March 2020, the Ministry of Health of the Government of the CoM made their “Plan of Action Against Coronavirus” public [3]. This included the intention to medicalize LTCHs and established that residents infected by SARS-CoV-2 would be treated at LTCHs. This plan was summarized in a press release; however, the full plan document was never made available to the general public nor to health professionals. In spite of this plan, reinforcement of LTCHs with medical staff and equipment did not take place.

On 18 March 2020, a ministerial directive was issued to public hospitals by the Director General of Social and Health Coordination of the CoM [4,5]. It requested hospitals to follow a protocol that included exclusion criteria barring hospital referrals for certain people living in LTCHs. Those requiring physical assistance due to moderate or severe disability according to the Barthel Index and those with moderate or severe cognitive impairment according to the Global Deterioration Scale were excluded from hospital referrals (Table 1).

In each hospital, liaison geriatricians were responsible for the triage of patients who were identified for a referral from LTCHs. These consultations were carried out by telephone and were initiated by LTCH staff.

On March 20, a version with modified exclusion criteria was issued by the CoM, and on March 24 and 25, further versions with new recommendations were issued (Table 1) [4,5,6]. These protocols were applied for several weeks until they fell out of use. No specific directives to halt the application of these successive protocols were issued.

### 1.4. Rationale and Aim of the Study

It is considered good medical practice to hospitalize a patient when the clinical judgment of the severity of the condition leads the doctor to believe that the patient will receive more adequate care at a hospital. Accordingly, the risk of mortality of COVID-19 patients referred to a hospital would be expected to be higher than that of COVID-19 patients remaining in LTCHs. This reasoning has been used in another study to assess the referral accuracy of primary care COVID-19 patients [7].

The aim of this study was to assess whether the CoM LTCH residents who had symptoms of respiratory infection and were excluded from hospital care by the administrative directive were more or equally likely to die than those who were referred to hospital, in comparison with similar LTCH cases from regions of Spain where such administrative directives were not issued. In epidemiological terms, our objective is to assess whether the hospitalization mortality ratio (HMR)—defined as the ratio of mortality in those referred to the hospital compared with the mortality of those who stayed at an LTCH—is greater than unity, as would be expected if most of the severe COVID-19 cases had been hospitalized.

## 2. Materials and Methods

The inclusion criteria were set to include published scientific articles or local government reports on mortality from COVID-19 infection in LTCHs in Spain between March and April 2020. We included all original reports published in journals and official epidemiological publications for each of the 17 regions of Spain that collected data to compute mortality ratios between COVID-19 patients transferred to hospital over those remaining in their LTCH.

Published articles were identified through a PubMed search with the following terms: “COVID”, “COVID-19”, or “SARS-CoV-2”; “mortality” or “fatality”; “long-term care” or “nursing home”; and “Spain”. Cross references were checked.

Of the 13 articles identified, six contained the data necessary to calculate the mortality ratio associated with hospital referral. In the other seven, the information was requested from the authors. Five authors were able to review medical records and determine deaths that occurred after hospital referral. Two authors replied that information on the place of death was not available in their databases. Finally, 11 studies were included. Their characteristics are shown in Table 2 [8,9,10,11,12,13,14,15,16,17,18,19,20].

An active search of official epidemiological publications for each of the 17 regions of Spain was undertaken. Two regions, Andalusia and Navarre, have published epidemiological data on COVID-19 mortality at LTCHs covering March and April 2020, but these reports do not contain information on the place of death. We requested this information through the *Portal de Transparencia* (Transparency Portal), a government office that provides access to information of public interest upon request. Andalusia provided the requested information. Data from Andalusia were included in this report [19]. In Navarre, the Department of Social Services did not have information on the place of death but suggested contacting the regional Public and Occupational Health Department, which was indeed able to supply data on all long-term care institutions for inclusion in this study [20].

The outcome of this study was the HMR. Other variables of interest for descriptive purposes were the percentage of hospitalizations and total mortality among COVID-19 cases.

For each study, the HMR was estimated at a 95% confidence interval, comparing the mortality percentage among residents with COVID-19 referred to a hospital with that of those who had remained at an LTCH.

**Figure 1:**
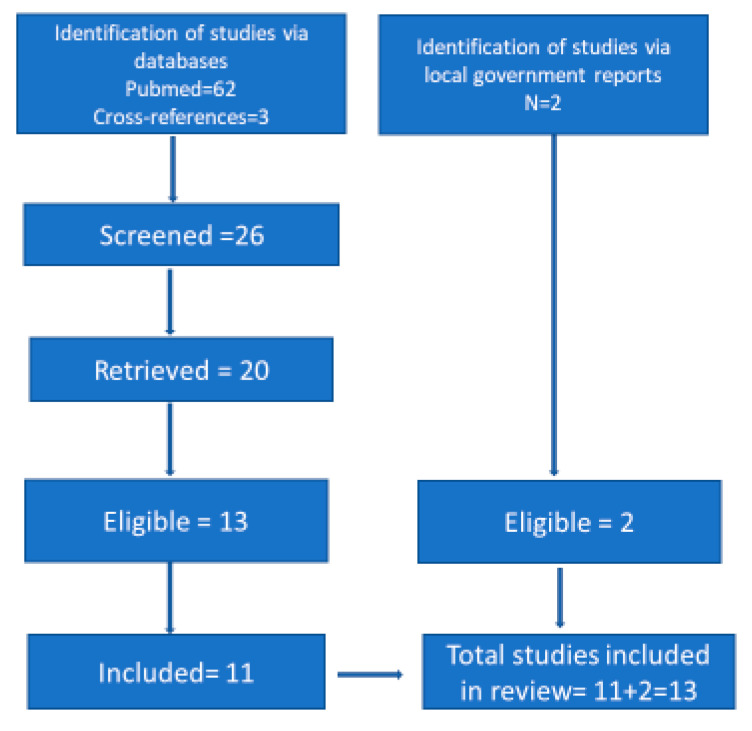
Flow diagram of the systematic review.

The first author (MVZ) reviewed the studies, extracting information from the published studies and email correspondence with the first authors of articles and reports as described above to complete Table 2 and Table 3. These emails are available upon request. Figure 1 shows the flow diagram of this systematic review, which followed the PRISMA 2020 guidelines for systematic reviews (Checklist tables in Supplementary Materials).

## 3. Results

### 3.1. Characteristics of the Studies

Table 2 shows the characteristics of the included studies. Two studies were conducted in the CoM and the remaining eleven were conducted in eight different regions: Andalusia, Aragon, the Basque Country, Castile-La Mancha, Catalonia, Galicia, Navarre, and the Valencian Community. Ten studies were based on retrospective cohorts, three were prospective cohorts, and only three studies included clinically compatible cases.

Three studies were conducted by a first author with an affiliation to a hospital geriatric department, two were conducted by hospital researchers, four by provincial or regional health departments, one by researchers at a public health research institution, one by researchers at a primary care service area, one by researchers at a university public health department, and one by an LTC provider. All studies include data from March and April 2020, but vary in length. Eleven studies include data collected up to the middle of June, covering the first epidemic wave. The remaining two studies include data up to December 2020 and January 2021, respectively [10,11]. The duration of cohort follow-up is shown in Table 2. Nine studies had a duration of less than 90 days. More than 60% of residents were moderately or severely disabled, either according to the Barthel Index or activities of daily living (ADL) dependency scale. More than half of the patients had cognitive scores indicative of moderate or severe cognitive impairment and more than 20% had a dementia diagnosis. The mean (or median) age was over 80 and more than two-thirds of the residents were women.

### 3.2. Hospital Referrals

**Figure 2:**
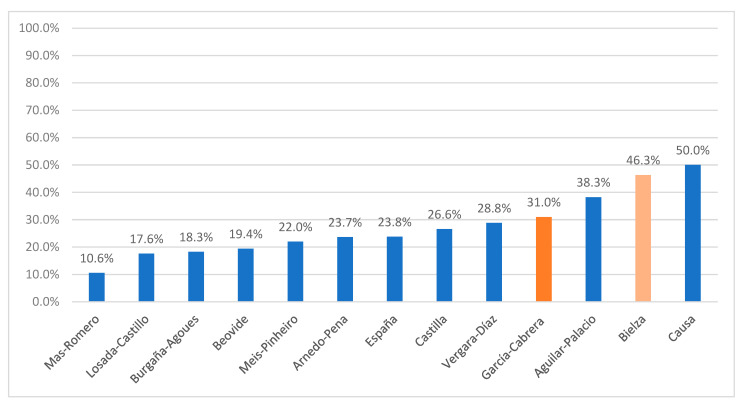
Percentage of hospitalizations of LTCH residents diagnosed with COVID-19 in the 13 reviewed studies.

Referrals from LTCHs to hospital were high in the two CoM hospital studies, with 46.3% in the study by Bielza et al., which included the month of May and therefore the waning of the first wave of the epidemic, and 31.0% in the study by García Cabrera et al., which covered the month of April. In the studies conducted outside of the CoM, referrals to hospitals were lower than 30%, with the exceptions of the small outbreak investigation conducted in Granada (where 50% of residents diagnosed with COVID-19 were hospitalized), and the study of all hospitals in the Autonomous Community of Aragon, which included the second and third wave of the epidemic (Figure 2).

### 3.3. Total Mortality

**Figure 3:**
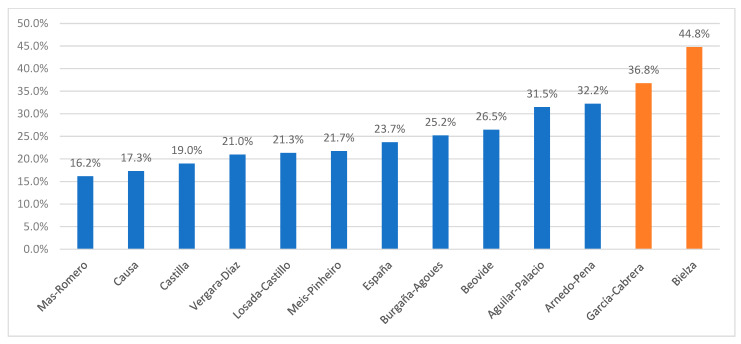
Percentage mortality of LTCH residents diagnosed with COVID-19 in the 13 reviewed studies.

Total mortality in residents diagnosed with COVID-19 in the studies of the two hospitals in the CoM (44.8 and 36.7%) was higher than in the studies conducted outside of the CoM, which had a range of between 16.2 and 32.2% (Table 3). It is worthy of note that the patients in the two outbreak investigations had the lowest mortality: 17.3 and 16.2% (Figure 3).

### 3.4. Mortality among Residents Who Remained at an LTCH

In the two studies conducted in the CoM, the mortality of COVID-19 patients who remained at an LTCH was 46.7 and 40.8%. In the studies outside the CoM, in-LTCH mortality varied between 25.9 and 7.7%. Here, it is again remarkable that the outbreak investigations showed low mortality for those who remained at the LTCHs: 7.7 and 11.9%. In Table 3, studies are ordered by mortality among those who stayed at the LTCH, labeled as “In-LTCH mortality N” (Table 3).

### 3.5. In-Hospital Mortality

In the study by García-Cabrera et al., in-hospital mortality was 27.7%, lower than the 42.5% in the Bielza study, which included the month of May when the first wave of the epidemic was waning, and the referral restrictions were no longer applied. Most of the studies conducted outside the CoM had high in-hospital mortality. For six of them, in-hospital mortality was higher than 50% (Table 3).

### 3.6. In-Hospital Mortality vs. In-LTCH Mortality

In-hospital mortality was significantly higher than mortality at LTCHs in eight of the ten studies conducted outside of the CoM (*p* < 0.05) (Table 3). In those eight studies, the mortality ratio was higher than 1 and ranged from 1.7 to 5. Confidence intervals included unity in one study with a mortality ratio of 1.4 (95%CI 0.9; 2.1), and in the very small study conducted in Granada, 3.5 (95%CI 0.8; 15.3).

The two studies carried out in the CoM differ. In the Bielza study, mortality did not vary according to the place of death, i.e., those who were referred to a hospital had a similar risk of death compared with those who remained at an LTCH. In the García-Cabrera study, those who were transferred to a hospital had a lower risk of death than those who were not referred. These different findings may be due to the fact that the Bielza study was extended until June 1. From mid-April onwards, the availability of hospital beds increased, and the hospital care restrictions were no longer in effect, and as a consequence, severe COVID-19 cases were more likely to be hospitalized. Consequently, this would shift the HMR towards values greater than unity. Indeed, in the Bielza study, the confidence interval for HMR includes unity, while in the shorter García-Cabrera study, the HMR has confidence interval limits lower than unity.

Concerning functional status, in the study by García-Cabrera et al., those who remained in an LTCH had worse ADL dependency and cognitive impairment indicators than those who were hospitalized. Similarly, in the study by Bielza et al., those who remained in an LTCH had greater frailty and dementia than hospitalized residents.

## 4. Discussion

### 4.1. Summary of Results

Hospitalizations were higher in the two studies conducted in the CoM compared with hospitalizations reported in other studies conducted in Spain during the first months of the epidemic.

Total mortality and mortality of COVID-19 residents who remained at an LTCH were higher in the studies conducted in the CoM than in the remaining studies. Lastly, in the studies conducted outside of the CoM, higher mortality was observed among those transferred to a hospital than among those who remained in LTCHs, with mortality ratios greater than unity. In the CoM, the mortality ratio for hospitalization was equal to or less than unity.

Our results support the assertion that the ministerial directive of the Government of the CoM could have been harmful to many LTCH residents because they were excluded from adequate hospital care based on their moderate or severe disability. In the studies conducted in the CoM, the poorer functional status of residents who remained at LTCHs, relative to the better functional status of those who were transferred to a hospital, could explain, at least partly, their higher mortality compared with residents who stayed at LTCHs outside of the CoM. The lack of staff due to sickness or fear of infection could have further aggravated the prognosis of those residents due to poor care, including lack of rehydration and nutrition.

### 4.2. Comparison with International Literature and Interpretation of Results

In a review of the international literature, we identified a local government directive to avoid hospital care for certain LTCH residents during the first wave of the COVID-19 epidemic in the province of Quebec (Canada) [21]. However, this government directive was not based on categorical criteria based on disability. In research conducted in 17 LTCHs in Greater Montreal, 1197 cases of confirmed COVID-19 infection were diagnosed. Following the government directive, only 63 were transferred to a hospital and 1134 were treated at an LTCH. Total mortality was very high (37.7%) and similar to what was reported in the CoM hospitals included in our research, but mortality was higher among the 63 COVID-19-infected residents transferred to a hospital (63.5%) than among those who remained at an LTCH (36.2%). The authors state that oxygen therapy was available in all the 17 LTCHs during the study period and, in fact, close to one-third of residents received it. We do not have quantitative data, but according to our knowledge based on interviews with nurses and medical doctors, oxygen therapy was unavailable in the LTCHs in the CoM between March and April 2020. Only sedatives were made gradually available to LTCHs during those months.

A study based on 1,319,839 electronic medical records of people aged 65 years and over residing in Catalonia and comparing COVID-19 mortality in institutionalized and non-institutionalized populations reported that neither age nor obesity was associated with COVID-19 mortality in LTCHs residents [22]. This suggests that LTCH residents were not treated in the same way as elderly persons not living in these institutions and supports claims by families of LTCH residents that access to hospital care was determined by place of residence. Notice that in our results the studies conducted in Catalonia have low HMR [13,18].

Paradoxically, the administrative directive issued to avoid unnecessary hospitalizations at a time of potential hospital collapse resulted in one of the highest hospitalization percentages of LTCH residents observed in the studies included in this review. A possible explanation would be that as hospital occupation dropped due to the waning of the epidemic in the general population around the second week of April, geriatricians were likely to hospitalize the more severe, but also some of the less severe COVID-19 diagnosed residents. This explanation is supported by the higher proportion of hospitalization observed in the Bielza study, which extended until the first of June, compared with the Garcia-Cabrera study, which ended on April 30. To test this hypothesis, individual patient electronic medical records would be needed.

The protocol issued by the Government of the CoM was not based on public health principles aimed at protecting vulnerable populations at higher risk of death [23] nor on face-to-face clinical assessments based on the severity of COVID-19 infection. It should be noted that many potential COVID-19 infections were not confirmed with a PCR test because those tests were not available at LTCHs, and consultations were carried out by phone.

The available data does not reveal the reasons that motivated the political decision to approve this disability-based triage protocol. Without any scientific basis, residents were selected according to functional status level. Residents with moderate and severe ADL dependency or cognitive impairment were kept in LTCHs, an environment with a high risk of infection and very limited healthcare personnel or health resources. Contrary to what occurred in Montreal, the medicalization of LTCHs announced by the CoM’s Ministry of Health on March 12th was never implemented. Private hospitals had available beds, oxygen therapy, and palliative care, but they were only offered to LTCH residents who had private insurance, as stated by the CEO of the second largest private healthcare company in Spain [24]. In addition, a temporary hospital with a capacity of more than 5000 beds was opened on March 21, but only 23 residents from LTCH homes were admitted, all of them in a single day, on March 26th. Moreover, 28% of LTCHs did not have a medical doctor, 22% had a doctor only on morning duty, and 22% of LTCH doctors were on leave of absence during those days [5].

We will never know what would have happened if the LTCHs had been reinforced with outbreak investigations, health professionals, and equipment, and if private hospitals and temporary hospitals had been used in addition to public ones to provide care for patients. The decrease in hospital referrals of infected patients might have occurred prior to the March 18 order, and most likely following the announcement of the intention to medicalize the LTCHs in the CoM made on March 12th, when the decision to care for LTCH residents diagnosed with COVID-19 was adopted. The government directive on March 18th might have been motivated by fear of hospital collapse and was sent to all hospitals to ensure that the exclusion criteria were applied in all referral requests initiated by LTCH staff.

### 4.3. Study Limitations

This review is based on studies covering different periods and regions. We may have right censoring because some of the LTCH residents diagnosed with COVID-19 who were alive at the end of the study period may have subsequently died. This censoring is likely to be larger during the first two months of the pandemic and smaller in the two studies covering the second half of 2020 when SARS-CoV-2 testing was available and hospital treatments were more effective. There is some risk of bias due to the possibility of missing further studies published in local government reports that we may have been unable to obtain.

We lack information on temporal changes in access to personal protective equipment (PPE), SARS-CoV-2 testing, and LTCH staff on sick leave. In fact, the increased availability of PPE and testing may have reduced staff sick leave, which, as a result, may have improved the quality of care at LTCHs. However, it is unlikely that these improvements in COVID-19 prevention and control at LTCHs would have had an effect on the decision to hospitalize residents because hospital referrals were decided by hospital geriatricians during phone consultations.

As stated above, no specific directives to halt the application of the disability-based triage were issued, but it seems that the triage was not applied from mid-April onwards, once it was established that COVID-19 mortality in the general population of the CoM was declining. According to a newspaper report, on April 10th, geriatricians had asked the Government of the CoM for a formal end to the administrative directive [25]. Once the exclusion protocols were no longer applied, hospital geriatricians decided on hospitalization following a medical exam in the context of the increasing availability of hospital beds [2]. This may have increased the hospitalization rates in the CoM as suggested by the comparison of hospitalizations in the two studies conducted in the CoM, because the study conducted during March and April had a lower hospitalization rate (31%) than the study extending through to the end of May (46.3%).

We lack official information on the population characteristics of LTCH residents in Spain. However, some of the cited research papers show that more than 75% of residents had a moderate or severe disability or cognitive impairment. The included studies covered very different populations. Four are based on the whole LTCH population of distinct autonomous regions of Spain (Andalusia, Aragon, the Basque Country, and Galicia). These are very large regions with thousands of LTCH residents and COVID-19 deaths. Other studies included all LTCHs in the referral area of one or two hospitals or a primary care district. Lastly, two small studies are based on outbreaks occurring in a single LTCH with referrals to a single hospital. The two hospitals in the CoM cited in this research are large and reported 1452 deaths out of the total 9468 deaths occurring in the LTCHs of the CoM during March and April 2020. However, findings are quite consistent across the studies in spite of the variation in the periods and regions. It is unlikely that these findings could be explained by strong confounders. The study in Montreal, serving as an international comparison of an area with a government directive to LTCHs that excluded the transfer of certain LTCH residents to hospital [18,26], was also large and based on 17 LTCHs and more than 1000 COVID-19-infected residents. However, in the Montreal case, the exclusion criteria were based on LTCH residents without severe COVID-19 symptomatology or where the necessary resources were available within the LTCH.

Lastly, the documents were reviewed by a single reviewer. However, the information in Table 2 and Table 3 could be verified by any interested reader.

## 5. Conclusions

The Government of the CoM’s hospital care exclusion protocol was applied to moderately and severely disabled elderly population living in LTCHs. It did not permit hospital referrals of many of the more severe cases that could have benefitted from hospital care, but rather referred those with better functional status.

Triage protocols should aim to save the maximum number of lives, be drawn up by multidisciplinary committees including experts on ethics, and be used only if there are no alternatives. Categorical exclusions should not be used [27,28]. Evaluation of the disability-based triage of LTCH residents during March–April 2020 in all public hospitals in the CoM should be conducted.

## Figures and Tables

**Table 1:** Rules and recommendations sent by the Government of the Community of Madrid to all public hospitals in the region in March 2020 concerning activities of daily living (ADL) dependency and cognitive impairment criteria to exclude LTCH residents from hospital care.

	Criteria for Hospital Referral of Patients with Respiratory Conditions:
**March 18**	**Hospital referral will proceed if the patient fulfills the following criteria:**
	Able to walk without assistance or has a Barthel Index > 60 No cognitive impairment or a Global Deterioration Scale (GDS) < 6 No advanced comorbidity
**March 20**	**Exclusion criteria for hospital referral of** **patients with respiratory conditions:**
	Neurodegenerative end of life (GDS = 7) Severe functional decline (Barthel Index < 25) Severe functional decline (Barthel Index 25–40) with moderate cognitive impairment (GDS = 5): ideally care should be provided at the LTCH
**March 24**	**Exclusion recommendations for hospital** **referral of patients with a respiratory** **condition:**
	Neurodegenerative end of life (GDS = 7) Severe functional decline (defined by Barthel Index < 25) Severe functional decline (Barthel Index 25–40) with moderate cognitive impairment (GDS = 5): ideally care should be provided at the LTCH, if this is not possible, refer to the hospital
**March 25**	**Recommendations for hospital referral of** **patients with a respiratory condition:**
	Taking into account the presence of symptoms, assessment of emergency care availability at the hospital, and prioritization of care at the LTCH, the patient should be transferred if they fulfill the following criteria after clinical assessment: Criteria of neurodegenerative end of life (GDS = 7) Frailty index => 7 (greater than or equal to 7)

**Table 2:** Characteristics of eligible studies.

First Author	Geographical Area	Confirmed/Clinical Cases	Period	No. of Hospitals in Geographical Area (GA)	Dependency	Cognitive Impairment
Bielza	CoM	Confirmed/clinical	20 March–1 June 2020	One	75%	50%
García Cabrera	CoM	Confirmed/clinical	30 March–30 April 2020	One	64%	44%
Aguilar-Palacio	Aragon	Confirmed	March 2020–March 2021	All in GA	UNK	36% dementia
Arnedo Pena	Castellon	Confirmed	March–May 2020	Two	78%	UNK
Beobide-Tellería	Guipuzcoa	Confirmed	March–December 2020	All in GA	75%	55%
Burgaña-Agoues	Cugat del Valles	Confirmed	15 March–15 May 2020	One	64%	72%
Causa	Granada	Confirmed	13 March–20 June 2020	One	60%	61%
España	Basque Country	Confirmed	February–22 May 2020	All in GA	UNK	33% dementia
Losada-Castillo	Galicia	Confirmed	1 March–12 June 2020	All in GA	UNK	UNK
Mas-Romero	Albacete	Confirmed/Clinical	6 March–5 April 2020	One	50%	22% dementia
Meis-Pinheiro	Catalonia	Confirmed	March–May 2020	Several	UNK	59% dementia
Vergara- Díaz	Andalusia	Confirmed	28 February–May 2020	All in GA	UNK	UNK
Castilla	Navarre	Confirmed	March–15 May 2020	All in GA	UNK	UNK

**Table 3:** Summary of studies of total COVID-19 mortality according to place of death of LTCH residents diagnosed with COVID-19, Spain 2020 *.

First Author	Geographical Area	COVID-19 Cases	Mortality N (%)	Hospitalization N (%)	In-Hospital Mortality N (%)	In-LTCH Mortality N (%)	Mortality Ratio (Hospital/ LTCH) (95% CI)
Bielza	CoM	630	282 (44.8%)	292 (46.3%)	124 (42.5%)	158 (46.7%)	0.9 (0.8; 1.1)
García-Cabrera		419	154 (36.7%)	130 (31.0%)	36 (27.7%)	118 (40.8%)	0.68 (0.5; 0.9)
Arnedo-Pena	Castellon	304	98 (32.2%)	72 (23.7%)	38 (52.8%)	60 (25.9%)	2.0 (1.5; 2.8)
Burgaña-Agoues	Sant Cugat del Valles	345	87 (25.0%)	63 (18.2%)	21 (33.3%)	66 (23.4%)	1.4 (0.9; 2.1)
Meis-Pinheiro	Catalonia	2092	455 (21.7%)	460 (22.0)	148 (32.1%)	307 (18.8%)	1.7 (1.4; 2.0)
Aguilar-Palacios **	Aragon	4632	1458 (31.5%)	1772 (38.3%)	935 (52.8%)	523 (18.3%)	2.9 (2.6; 3.2)
Beobide **	Guipuzcoa	170	45 (26.4%)	33 (19.4%)	22 (66.6%)	23 (16.8%)	4.0 (2.5; 6.2)
España	Basque Country	2140	507 (23.7%)	510 (23.8%)	257 (50.4%)	250 (15.3%)	3.3 (2.8; 3.8)
Castilla	Navarre	1483	301 (20.3%)	429 (28.9%)	155 (36.1)	146 (13.8%)	2.6 (2.1; 3.2)
Mas-Romero	Albacete	198	32 (16.2%)	21 (10.1%)	11 (52.4%)	21 (11.9%)	4.4 (2.5; 7.8)
Losada- Castillo	Galicia	1276	272 (21.3%)	225 (17.6%)	141 (62.7%)	131 (12.5%)	5.0 (4.2; 6.1)
Vergara- Díaz	Andalusia	2581	541 (21.0%)	744 (28.8%)	340 (45.7%)	201 (10.9%)	4.2 (3.6; 4.9)
Causa	Granada	52	9 (17.3)	26 (50%)	7 (26.9%)	2 (7.7%)	3.5 (0.8; 15.3)

* Those who died in transit or at an intermediate healthcare center are classed as hospital deaths. ** These studies report deaths within 90 days of diagnosis and extend until March 2021 (Aguilar-Palacios) and December 2020 (Beobide).

## Data Availability

No new data were created because this research is based on published studies. In some cases, additional information was required from the first authors. The original correspondence is available upon request.

## Supplementary Materials

The following supporting information can be downloaded at: https://www.mdpi.com/article/10.3390/epidemiologia4020019/s1, Table S1. PRISMA 2020 checklist; Table S2. PRISMA 2020 for abstract checklist. Word limit in epidemiology: 150 words.

## References

[1] Zunzunegui M.V., Rico M., Béland F., García-López F.J. (2022). The Impact of Long-Term Care Home Ownership and Administration Type on All-Cause Mortality from March to April 2020 in Madrid, Spain. Epidemiologia.

[2] Condes E., Arribas J.R. (2021). Impact of COVID-19 on Madrid hospital system. Enferm. Infecc. Microbiol. Clin..

[3] La Comunidad de Madrid Pone en Marcha un Plan Histórico que Unirá la Sanidad Pública y Privada Bajo una Única Coordinación. https://www.comunidad.madrid/notas-prensa/2020/03/12/comunidad-madrid-pone-marcha-plan-historico-unira-sanidad-publica-privada-unica-coordinacion.

[4] Rico M. (2020). Los Seis Documentos que Demuestran que Ayuso Miente Sobre la Orden de no Trasladar Enfermos de Residencias a Hospitales. Infolibre. https://www.infolibre.es/politica/seis-documentos-demuestran-ayuso-miente-orden-no-trasladar-enfermos-residencias-hospitales_1_1183785.html.

[5] Reyero Zubiri A. (2022). Morirán de Forma Indigna.

[6] Rico M. (2021). Verguenza: El Escándalo de las Residencias.

[7] Guzmán-García M.B., Mohedano-Moriano A., González-González J., Morales-Cano J.M., Campo-Linares R., Lozano-Suárez C., Estrada-Álvarez T.P., Romero-Fernández M.M., Aguilar-Galán E.V., Criado-Álvarez J.J. (2022). Lung Ultrasound as a Triage Method in Primary Care for Patients with Suspected SARS-CoV-2 Pneumonia. J. Clin. Med..

[8] Bielza R., Sanz J., Zambrana F., Arias E., Malmierca E., Portillo L., Thuissard I.J., Lung A., Neira M., Moral M. (2021). Clinical Characteristics, Frailty, and Mortality of Residents With COVID-19 in Nursing Homes of a Region of Madrid. J. Am. Med. Dir. Assoc..

[9] García-Cabrera L., Pérez-Abascal N., Montero-Errasquín B., Rexach Cano L., Mateos-Nozal J., Cruz-Jentoft A. (2021). Characteristics, hospital referrals and 60-day mortality of older patients living in nursing homes with COVID-19 assessed by a liaison geriatric team during the first wave: A research article. BMC Geriatr..

[10] Aguilar-Palacio I., Maldonado L., Marcos-Campos I., Castel-Feced S., Malo S., Aibar C., Rabanaque M. (2022). Understanding the COVID-19 Pandemic in Nursing Homes (Aragón, Spain): Sociodemographic and Clinical Factors Associated with Hospitalization and Mortality. Front. Public Health.

[11] Arnedo-Pena A., Romeu-García M.A., Gascó-Laborda J.C., Meseguer-Ferrer N., Safont-Adsuara L., Prades-Vila L., Flores-Medina M., Rusen V., Tirado-Balaguer M.D., Sabater-Vidal S. (2022). Incidence, Mortality, and Risk Factors of COVID-19 in Nursing Homes. Epidemiologia.

[12] Beobide Telleria I., Ferro Uriguen A., Laso Lucas E., Sannino Menicucci C., Enriquez Barroso M., López de Munain Arregui A. (2022). Risk factors associated with COVID-19 infection and mortality in nursing homes. Aten. Primaria.

[13] Burgaña Agoües A., Serra Gallego M., Hernández Resa R., Joven Llorente B., Lloret Arabi M., Ortiz Rodriguez J., Acebal H.P., Hernández M.C., Ayala I.C., Calero P.P. (2021). Risk factors for COVID-19 morbidity and mortality in institutionalised elderly people. Int. J. Environ. Res. Public Health.

[14] Causa R., Nievas D.A., Bermúdez Tamayo C. (2021). COVID-19 y dependencia funcional: Análisis de un brote en un centro sociosanitario de personas mayores. Rev. Esp. Salud Pública.

[15] España P.P., Bilbao A., García-Gutiérrez S., Lafuente I., Anton-Ladislao A., Villanueva A., Uranga A., Legarreta M.J., Aguirre U., Quintana J.M. (2021). Predictors of mortality of COVID-19 in the general population and nursing homes. Intern. Emerg Med..

[16] Losada-Castillo I., Santiago-Pérez M.I., Naveira-Barbeito G., Otero-Barros M.T., Pérez-Martínez O., Zubizarreta-Alberdi R. (2022). Impact of COVID-19 pandemic in terms of incidence and lethality in nursing homes in Galicia (Spain). Rev. Esp. Geriatr. Gerontol..

[17] Mas Romero M., Avendaño Céspedes A., Tabernero Sahuquillo M.T., Cortés Zamora E.B., Gómez Ballesteros C., Sánchez-Flor Alfaro V., Bru R.L., Utiel M.L., Cifuentes S.C., Longobardo L.M.P. (2020). COVID-19 outbreak in long-term care facilities from Spain. Many lessons to learn. PLoS ONE.

[18] Meis-Pinheiro U., Lopez-Segui F., Walsh S., Ussi A., Santaeugenia S., García-Navarro J.A., San-Jose A., Andreu A.L., Campins M., Almirante B. (2021). Clinical characteristics of COVID-19 in older adults. A retrospective study in long-term nursing homes in Catalonia. PLoS ONE.

[19] Vergara-Diaz A., Forcada Falcón M. (2020). Análisis de Brotes Declarados en Residencias de Mayores por Coronavirus SARS-CoV-2 en Andalucía 2020. Sistema de Vigilancia Epidemiológica de Andalucía.

[20] Castilla J. (2020). Vigilancia Epidemiológica en Navarra. http://www.navarra.es/NR/rdonlyres/3855C06B-B6A2-4E05-BA57-034AB7947A04/477682/InformesEpidemiologicos_2020pp.pdf.

[21] Zhang X.S., Charland K., Quach C., Nguyen Q.D., Zinszer K. (2022). Institutional, therapeutic, and individual factors associated with 30-day mortality after COVID-19 diagnosis in Canadian long-term care facilities. J. Am. Geriatr. Soc..

[22] Marin-Gomez F.X., Mendioroz-Peña J., Mayer M.A., Méndez-Boo L., Mora N., Hermosilla E., Coma E., Vilaseca J.-M., Leis A., Medina M. (2022). Comparing the clinical characteristics and mortality of residential and non-residential older people with COVID-19: Retrospective observational study. Int. J. Environ. Res. Public Health.

[23] Ioannidis J.P.A. (2021). Precision shielding for COVID-19: Metrics of assessment and feasibility of deployment. BMJ Glob. Health.

[24] Fernández M. Pelea por Dinero en la Sanidad Privada. https://elpais.com/economia/2020-04-17/pelea-por-dinero-en-la-sanidad-privada.html.

[25] Peinado F. Los Días Más Duros de los Geriatras de Madrid. https://elpais.com/espana/madrid/2020-06-12/los-dias-mas-duros-de-los-geriatras-de-madrid.html.

[26] Guide Pour la Prise en Charge des Résidents en Centres D’hébergement et de Soins de Longue Durée (CHSLD) Dans le Contexte de la Pandémie de la COVID-19. https://santesaglac.gouv.qc.ca/medias/2020/04/20-MS-02502-61_Guide_CHSLD_13_avril.pdf.

[27] Rueda J. (2021). Ageism in the COVID-19 pandemic: Age-based discrimination in triage decisions and beyond. Hist. Philos. Life Sci..

[28] Scully J.L. (2020). Disability, Disablism, and COVID-19 Pandemic Triage. Bioethical Inq..

