# Supplementary material for: Restrictions on Hospital Referrals from Long-Term Care Homes in Madrid and COVID-19 Mortality from March to June 2020: A Systematic Review of Studies Conducted in Spain
Source: Epidemiologia (Basel). 2023 Jun 6;4(2):176–87. doi: 10.3390/epidemiologia4020019 (PMC10296840; doi:10.3390/epidemiologia4020019)
Supplement: Supplementary file 1 [file epidemiologia-04-00019-s001.zip › epidemiologia-2243455-supplementary.pdf]

Supplementary material

Table S1. PRISMA 2020 checklist

Section and topic	Item #	Location where item is reported
**Title**		
Title	1	Lines 2 and 3
**Abstract**		
Abstract	2	See Table S2
**Introduction**		
Rationale	3	Lines 26-56 and 80-85
Objective	4	Lines 87-95
**Methods**		
Eligibility criteria	5	Lines 97-102. We applied inclusion criteria. No exclusion criteria was applied.
Information sources	6	99-102. Figure 1.
Search strategy	7	Lines 103-105 and 112-113. Figure 1.
Selection process	8	Lines 106-108 and 113-114. Figure 1.
Data collection process	9	Lines 106-110, 115-117
Data items	10a	Line 123
	10b	Lines 124-125
Study risk of bias assessment	11	Lines 129-131
Effect measures	12	Line 126-128
Synthesis methods	13a	Table 2. Line 145
	13b	Lines 110-111
	13c	Line 121
	13d	Lines 126-128. Table 3.
	13e	Lines 149-162
	13f	No sensitivity analyses was conducted
Reporting bias assessment	14	Lines 112-113
Certainty assessment	15	Line 126
**Results**		
Study selection	16a	Table 2. Line 145
	16b	All identified studies fulfill selection criteria based on inclusion criteria
Study characteristics	17	Table 1

Risk of bias in studies	18	Lines 142-143
Results of individual studies	19	Table 3. Line 196<br>Figures 2 and 3
Results of synthesis	20a	One outcome in Column 8th of Table 3.
	20b	Table 3.
	20c	No heterogeneity analyses were conducted
	20d	No sensitivity analyses.
Reporting biases	21	Potential bias discussion in lines 321-328
Certainty of evidence	22	Table 3, Column 8th
Discussion		
Discussion	23a	Lines 257-289
	23b	Lines 321-369
	23c	Line 326-328 and 366-367
	23d	Lines 369-377
Other information	24a	The review was not registered
	24b	The review protocol was not published
	24c	Not apply
Support	25	No financial support was available
Competing interest	26	None
Availability of data code and other materials	27	All data is publicly available.

Table S2. PRISMA 2020 for abstract checklist. Word limit in epidemiology: 150 words

Section and topic	Item #	Checklist item
**Title**	1	x
**Background**		
Objectives	2	x
**Methods**		
Eligibility criteria	3	x
Information sources	4	x
Risk of bias	5	Not covered in abstract. The abstract word number is 150
Synthesis of results	6	x
**Results**		
Included Studies	7	x
Synthesis of results	8	x
**Discussion**		
Limitations of evidence	9	X
Interpretation	10	X
**Other**		
Funding	11	At the end of the main paper
Registration	12	Not applied
